# AI-Based Noninvasive Blood Glucose Monitoring: Scoping Review

**DOI:** 10.2196/58892

**Published:** 2024-11-19

**Authors:** Pin Zhong Chan, Eric Jin, Miia Jansson, Han Shi Jocelyn Chew

**Affiliations:** 1 Department of Nursing Ng Teng Fong General Hospital Singapore Singapore; 2 Yong Loo Lin School of Medicine National University of Singapore Singapore Singapore; 3 Research Unit of Health Sciences and Technology University of Oulu Oulu Finland; 4 Alice Lee Centre for Nursing Studies Yong Loo Lin School of Medicine National University of Singapore Singapore Singapore

**Keywords:** artificial intelligence, blood glucose, diabetes, noninvasive, self-monitoring, machine learning, scoping review, monitoring, management, health informatics, deep learning, accuracy, heterogeneity, mobile phone

## Abstract

**Background:**

Current blood glucose monitoring (BGM) methods are often invasive and require repetitive pricking of a finger to obtain blood samples, predisposing individuals to pain, discomfort, and infection. Noninvasive blood glucose monitoring (NIBGM) is ideal for minimizing discomfort, reducing the risk of infection, and increasing convenience.

**Objective:**

This review aimed to map the use cases of artificial intelligence (AI) in NIBGM.

**Methods:**

A systematic scoping review was conducted according to the Arksey O’Malley five-step framework. Eight electronic databases (CINAHL, Embase, PubMed, Web of Science, Scopus, The Cochrane-Central Library, ACM Digital Library, and IEEE Xplore) were searched from inception until February 8, 2023. Study selection was conducted by 2 independent reviewers, descriptive analysis was conducted, and findings were presented narratively. Study characteristics (author, country, type of publication, study design, population characteristics, mean age, types of noninvasive techniques used, and application, as well as characteristics of the BGM systems) were extracted independently and cross-checked by 2 investigators. Methodological quality appraisal was conducted using the Checklist for assessment of medical AI.

**Results:**

A total of 33 papers were included, representing studies from Asia, the United States, Europe, the Middle East, and Africa published between 2005 and 2023. Most studies used optical techniques (n=19, 58%) to estimate blood glucose levels (n=27, 82%). Others used electrochemical sensors (n=4), imaging (n=2), mixed techniques (n=2), and tissue impedance (n=1). Accuracy ranged from 35.56% to 94.23% and Clarke error grid (A+B) ranged from 86.91% to 100%. The most popular machine learning algorithm used was random forest (n=10) and the most popular deep learning model was the artificial neural network (n=6). The mean overall checklist for assessment of medical AI score on the included papers was 33.5 (SD 3.09), suggesting an average of medium quality. The studies reviewed demonstrate that some AI techniques can accurately predict glucose levels from noninvasive sources while enhancing comfort and ease of use for patients. However, the overall range of accuracy was wide due to the heterogeneity of models and input data.

**Conclusions:**

Efforts are needed to standardize and regulate the use of AI technologies in BGM, as well as develop consensus guidelines and protocols to ensure the quality and safety of AI-assisted monitoring systems. The use of AI for NIBGM is a promising area of research that has the potential to revolutionize diabetes management.

## Introduction

According to the International Diabetes Federation, around 537 million adults aged 20-79 years were diagnosed with diabetes in 2021, a number that has been projected to increase to 783 million in 2045 [1]. Chronic diabetes mellitus (DM) leads to many severe complications, including stroke, blindness, ulcers, kidney failure, and vascular damage [2]. DM management places a massive burden on health care expenditure, which has more than quadrupled to at least US $966 billion over the last 15 years [1]. The most common and possibly life-threatening complication of DM is hypoglycemia [3], where common symptoms include autonomic (anxiety, tremors, palpitations, and diaphoresis) and neuroglycopenic (blurred vision, dizziness, headache, and loss of consciousness) manifestations [4]. Therefore, individuals with DM are often advised to monitor their blood glucose levels regularly to detect and manage abnormalities [4]. However, current blood glucose monitoring (BGM) methods are often invasive and require repetitive pricking of a finger to obtain blood samples, predisposing individuals to pain, discomfort, and infection [5]. The threshold for the onset of hypoglycemia also differs among patients (ie, typically higher in patients with uncontrolled diabetes), indicating the need for personalized BGM strategies [4].

Besides invasive BGM techniques, minimally invasive and noninvasive techniques have been developed. The most common minimally invasive method adopts the glucose-oxidase principle where a wire-based sensor is inserted in the subcutaneous layer of the skin [6]. It involves a calibration process that measures the current signal from the interstitial fluid rather than from the blood [7]. However, frequent calibration is required to maintain sensor accuracy by using traditional invasive fingerpick samples as a reference [8]. Recent flash glucose monitoring uses factory calibration which does not require calibration by the user but this method requires frequent replacement of the needle electrode every 1-2 weeks [9].

Noninvasive blood glucose monitoring (NIBGM) is ideal for minimizing discomfort, reducing the risk of infection, and increasing convenience. The latest advancements include near-infrared spectroscopy (NIRS), photoplethysmography (PPG), Raman spectroscopy (RS), photoacoustic signals, and biosensors, like saliva and tears [10]. As noninvasive methods do not directly detect blood glucose levels from blood samples, artificial intelligence (AI) could be used to estimate and predict blood glucose levels based on specific features selected. The use of AI could also facilitate the personalized BGM to inform treatment options, including insulin initiation and titration [11,12]. Although AI algorithms have been widely used in various health care settings including decision support systems and warning systems for hypoglycemia in patients with T1DM, little is known regarding the applicability of noninvasive methods [13,14].

Several studies have explored noninvasive methods for measuring and monitoring blood glucose levels in patients with and those without DM [15-17]. However, these reviews did not cover the use of machine learning (ML) systems often embedded in these devices, nor did they perform a comprehensive analysis of the accuracy of the devices. Similarly, some reviews have focused on the use of AI approaches for diabetes diagnosis and management using optical sensors [17] and breath analysis [18]. While these reviews present a comprehensive analysis of the available and used ML models, they often only cover one method of data collection, such as optical sensors. Two reviews focused on heart rate variability analysis [19,20] while another review focused on both electrocardiography (ECG) and PPG signals [21]. Furthermore, another review focused on detailing an overview of ML and AI techniques in the field of DM detection and self-management but not on NIBGM [22]. Therefore, a comprehensive review of the existing literature is needed to understand the current status of the use of AI in NIBGM. Given the novelty of using AI in NIBGM systems, evidence on the accuracy and effectiveness of such technologies is limited. Thus, we conducted a scoping review to rapidly map the key concepts and evidence regarding the use of AI for continuous NIBGM. Our findings would scope the available evidence on this topic, and identify the existing research gaps to inform the value and direction of conducting a full systematic review [23].

## Methods

### Overview

This scoping review was conducted using Arksey and O’Malley’s five-step framework and reported according to the PRISMA-ScR (Preferred Reporting Items for Systematic Reviews and Meta‐Analyses extension for Scoping Reviews) guidelines (Multimedia Appendix 1) [24].

### Step 1: Identifying the Research Question

Our primary research question was as follows: (1) what are the use cases of AI-assisted noninvasive BGM systems? Our secondary research question was as follows: (2) what are the AI models developed for noninvasive BGM?

### Step 2: Identifying Relevant Studies

A comprehensive literature search was conducted on 08 February 2023 across 8 major databases, namely CINAHL, Embase, PubMed, Web of Science, Scopus, The Cochrane-Central Library, ACM Digital Library, and IEEE Xplore. The following key terms were used in the search: “glucose monitoring”; “monitoring glucose”; “artificial intelligence”; “computer heuristics”; “fuzzy logic”; “knowledge bases”; “machine learning”; “natural language processing”; “neural networks”; and “sentiment analysis” (Multimedia Appendix 2). Content experts were consulted and previous reviews on similar topics were hand searched for additional relevant studies.

### Step 3: Study Selection and Methodological Quality Assessment

After the search was completed, duplicate studies were identified and removed. The remaining papers had their abstracts screened in a double-blinded, independent manner by 2 investigators (PZC and HSJC) according to the following inclusion criteria: prospective and retrospective primary studies that described the use of AI for continuous noninvasive BGM among human participants and written primarily in English. Papers were excluded if they: (1) were reviews or gray literature, (2) did not involve AI in the continuous NIBGM, (3) involved nonhuman participants, or (4) were primarily non–English-language papers. Disputes were resolved via discussion and consensus by the 2 investigators. Both investigators (PZC and HSJC) then combed the relevant journals, bibliography, and conference submissions to identify more relevant papers, and all papers then underwent a full-text sieve based on the inclusion and exclusion criteria.

The methodological quality appraisal of each study was conducted independently by 2 reviewers (PZC and EJ) using the Checklist for assessment of medical AI (ChAMAI) [25]. Reviewers rated each included study on 30 items representing 6 domains namely problem understanding, data understanding, data preparation, modeling, validation, and deployment. Each item (bolded to represent having a high priority) received a rating of OK (adequately addressed), mR (sufficient but improvable), or MR (inadequately addressed, corresponding to a score of 2, 1, and 0, respectively. Items on a low-priority (not bolded) received half the scores and the maximum total score is 50 [26]. Overall scores indicate the study quality to be low (0-19.5), medium (20-34.5), or high (35-50). Discrepancies were resolved by a third reviewer (HSJC).

### Step 4: Data Charting

Data were extracted independently and cross-checked by 2 investigators (PZC and HSJC). Any disputes were resolved via discussion and consensus. Study characteristics extracted included the author names, country, type of publication, study design, population characteristics, mean age, types of noninvasive techniques used, and application, as well as characteristics of the BGM systems (use of AI, AI type, AI features, types of data imputed, technology used, dataset, validation, proportion of training and testing dataset, and metrics used).

## Results

### Step 5: Collating, Summarizing, and Reporting the Results

**Figure 1:**
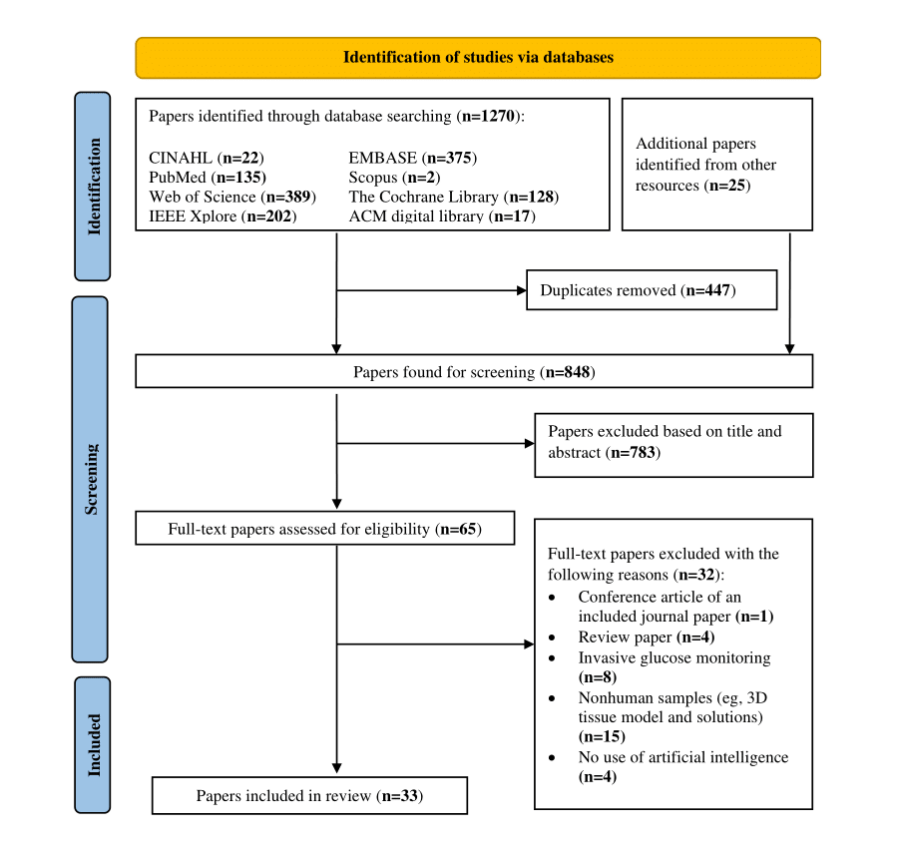
PRISMA-ScR flowchart. PRISMA-ScR: Preferred Reporting Items for Systematic Reviews and Meta‐Analyses extension for Scoping Reviews.

Our initial search yielded 1270 studies. After removing duplicated citations, screening through 848 titles and abstracts, and 65 full-text papers, 33 papers were included in this scoping review (Figure 1).

### Study Characteristics

Most of the included studies were conducted in Asia (n=21, 64%) [10,27-46], were peer-reviewed journal papers (n=24, 73%) [10,30-41,47-57], were prospective cohort studies (n=20, 61%) [30-33,35-43,47,49,51-53,57,58], and used optical (eg, near-infrared and PPG) techniques (n=19, 58%) [10,27,29,31,33,35-37,39,40,42,44,45,47,51,53,56-58] to estimate blood glucose levels (n=27, 82%) [27,29-31,33-38,40-43,45-48,50-53,55-58] (Table 1). Most of the studies did not report the population characteristics [10,27,29,33,35-39,41,42,44-46,48,54,56-58], mean age [10,27-36,39,41-43,45,46,48,50-53,55,57], and sex [27-29,33,35,36,38-41,43-48,50-52,54,55,57,58].

**Table 1:** Summary of study characteristics.

Study characteristics		Values (N=33), n (%)
**Country**		
	Algeria [48]	1 (3)
	Bangladesh [27]	1 (3)
	China [33,35,39-41]	5 (15)
	China (Hong Kong) [30]	1 (3)
	India [10,31,32,34,43-45]	7 (21)
	Indonesia [42]	1 (3)
	Israel [37]	1 (3)
	Malaysia [46]	1 (3)
	Mexico [47,58]	2 (6)
	Netherlands [52]	1 (3)
	South Korea [36,38]	2 (6)
	Spain [56]	1 (3)
	Sri Lanka [28,29]	2 (6)
	United States [49-51,53-55,57]	7 (21)
**Type of publication**		
	Journal paper [10,30-41,47-57]	24 (73)
	Conference papers [27-29,42-46,58]	9 (27)
**Study design**		
	Prospective cohort [30-33,35-43,47,49,51-53,57,58]	20 (61)
	Retrospective cohort [10,28,44,48,50,54,56]	7 (21)
	Others [27,29,34,45,46,55]	6 (18)
**Population characteristics**		
	Type 1 DM^a^ [50,52]	2 (6)
	Type 2 DM [28]	1 (3)
	Healthy [47,51]	2 (6)
	Mixture [30-32,34,40,43,49,53,55]	9 (27)
	NR^b^ [10,27,29,33,35-39,41,42,44-46,48,54,56-58]	19 (58)
**Age (years)**		
	21-40 [38,44,47,54,56,58]	6 (18)
	40-65 [37,40,49]	3 (9)
	NR [10,27-36,39,41-43,45,46,48,50-53,55,57]	24 (73)
**Sex (male)**		
	0 [10]	1 (3)
	<50 [34,49]	2 (6)
	>50 [30-32,37,42,53,56]	7 (21)
	NR [27-29,33,35,36,38-41,43-48,50-52,54,55,57,58]	23 (69.70)
**Noninvasive techniques**		
	Optical (NIR^c^, PPG^d^, Raman) [10,27,29,31,33,35-37,39,40,42,44,45,47,51,53,56-58]	19 (58)
	Impedance [55]	1 (3)
	Biosensor (breath, saliva, tears) [30,32,34,52]	4 (12)
	Imaging (ECG^e^, UWB^f^) [46,48]	2 (6)
	Mixture [38,50]	2 (6)
	NR [28,41,43,49,54]	5 (15)
**Applications**		
	Predict DM [10,44]	2 (6)
	Monitoring by physician [43,53]	2 (6)
	Estimate BG^g^ levels [10,27,29-42,45-48,50-52,54-58]	27 (82)
	Estimate HbA_1c_^h^ levels [49]	1 (3)
	Predict future BG levels [28]	1 (3)

^a^DM: diabetes mellitus.

^b^NR: not reported.

^c^NIR: near-infrared.

^d^PPG: photoplethysmography.

^e^ECG: electrocardiography.

^f^UWB: ultrawideband.

^g^BG: blood glucose.

^h^HbA_1c_: hemoglobin A_1c_.

### Use Cases of AI-Assisted NIBGM

The majority of the use cases were to estimate blood glucose levels (n=29, 88%), [10,27,29-31,33-38,40-48,50-53,55-58], 3 (9%) were to detect DM [10,32,54], 1 (3%) was to estimate suitable insulin doses [44], and another was to predict future blood glucose level (Table 1 and Multimedia Appendix 3 [10,27-58]). Only one study used AI-assisted NIBGM to estimate hemoglobin A_1c_ (HbA_1c_) levels and blood glucose variability among adults with prediabetes [49], which is noteworthy as glucose variability refers to oscillations in blood glucose levels throughout the day and could suggest the severity of diabetic complications.

### BGM Technology

**Figure 2:**
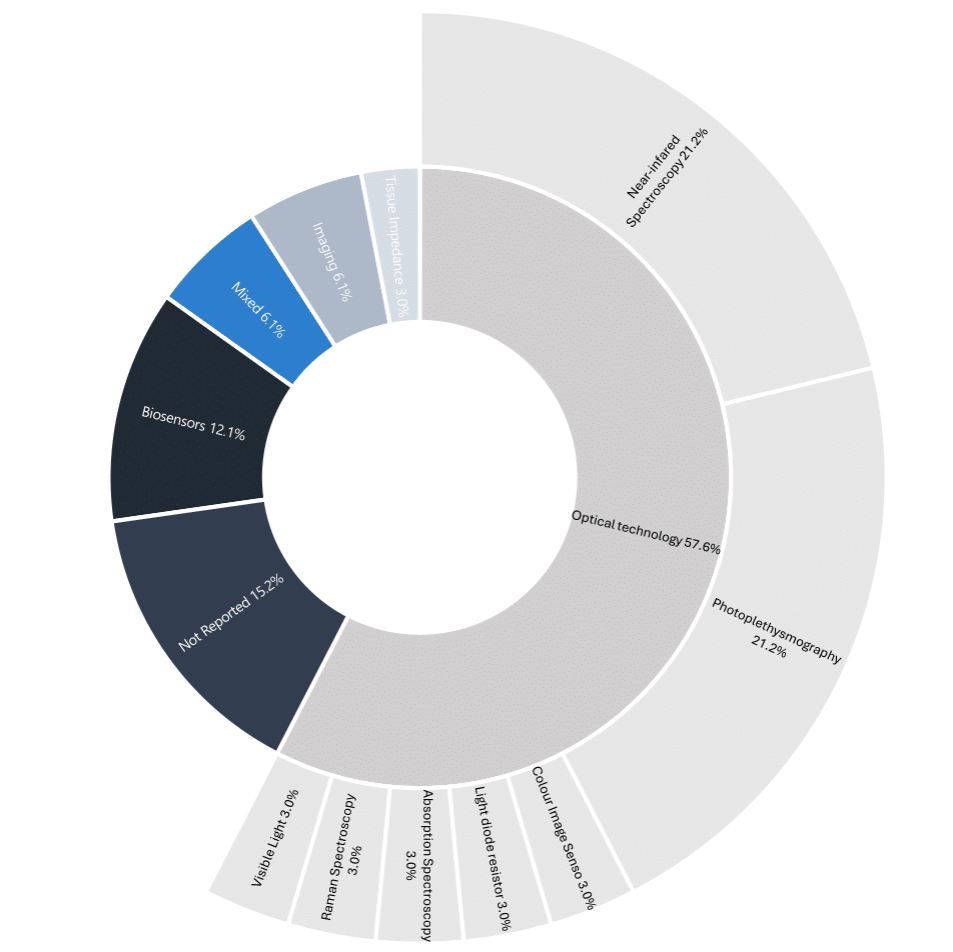
Blood glucose monitoring technology.

A summary of the technology used for NIBGM is shown in Multimedia Appendices 4 and 5 [10,27-58] and Figure 2. Of the 33 studies, 19 studies experimented with devices to estimate blood glucose levels using optical methods including PPG (n=7, 21%) [27,31,35,40,44,45,56], NIRS (n=7, 21%) [10,29,36,39,42,53,57], RS (n=1, 3%) [51], absorption spectroscopy (n=1, 3%) [33], noninvasive optical analysis of visible light capture by specialized cameras (n=1, 3%) [47], color image sensor (n=1, 3%) [37], and laser beam and light diode resistor (n=1, 3%) [58], 4 studies used devices that detected biological substances, including biosensor for tear glucose [37] and breath analysis [48] (two were not reported [30,52]), 3 (6%) studies used imaging techniques, including ECG [48] and ultrawideband (UWB) [46], 2 studies used mixed methods, including Optical+Electromagnetic+Thermal techniques [36] and Impedance+Multi-Wavelength NIR Spectroscopy [38], and 1 study used a device that measured tissue impedance (Multimedia Appendix 4 [10,27-58]). NIR spectroscopy detects the intensity of the reflected near-infrared light by glucose molecules in the blood to estimate blood glucose levels [35]. PPG operates on the same principles as that of the pulse oximeter, by calculating blood glucose levels based on the light intensity detected on a receiver and sent out by a transmitter [12]. RS works by comparing the Raman light emitted from a scattering medium (tissue) for transcutaneous determination of compositions of molecules, such as glucose, in the tissue-blood matrix [15,51]. RS can noninvasively monitor variations in glucose present at low concentrations in the blood-tissue matrix of the skin due to its distinct characteristic spectral features.

NIBGMs based on biosensors use breath, saliva, or tear samples to derive blood glucose concentrations based on their components, such as sodium, potassium, and calcium ions. ECGs measure the electrical activities of the heart and present them as a PQRST wave, with various abnormalities of the waves seemingly correlated with hyper- and hypoglycemia [49]. UWB imaging estimates blood glucose change via changes in the blood dielectric properties [46]. Two devices had mixed methods of detection. The device by Song et al [38] used both impedance and NIR to estimate blood glucose levels, while the device by Abubeker and Baskar [44] integrated ultrasonic, electromagnetic, and thermal data from the patient. Finally, the device by Malinin et al [55] measured the impedance of tissues via bracelet-type electrodes to detect blood glucose levels by monitoring the transfer functions of a tissue segment in the electromagnetic field.

A total of 19 (58%) devices used light-related signals as the input data (PPG signals [27,31,35,36,40,45,56], UWB imaging [46], NIR signals [10,29,39,53,57], Raman Spectra [51], nonvisible light signals [33,37,58], visible light signals [47], and LED light [42]), 5 (15%) devices collected biological samples (tears [52,59], breath [30,32], and saliva [34]), 4 (12%) devices used images or videos (video of finger [27,40], facial video [35], and image of finger or ear [57]), 4 (12%) devices collected vitals (eg, oxygen saturation, heart rate, and skin temperature [41,44,49,50]), 1 (3%) device collected impedance data [55], 1 (3%) device used ECG data [48], 1 (3%) device used a combination of types of inputs (intensity modulated photocurrent spectroscopy and multiwavelength NIRS) [38], 2 (6%) devices extracted various attributes from the patient’s lifestyle and background, with one using medication intake, food intake, daily activities, and measured blood glucose levels as the input [28], and the other using pregnancy, BMI, insulin level, age, blood pressure, skin thickness, glucose, and diabetes pedigree function [54].

### AI Models Developed for NIBGM

A summary of the characteristics of ML is shown in Multimedia Appendix 5 [10,27-58]. The accuracy of NIBGM estimating blood glucose ranged from 35.56% [38] to 94.23% [38], mean absolute error (MAE) ranged from 0.248 [54] to 11.8 [15], *R*^2^ ranged from 0.11 [44] to 0.91 [37], and Clarke error grid (CEG; A+B) ranged from 86.91% [50] to 100% [12,15,23,25,36,37,39,41]. Both MAE and *R*^2^ were used to evaluate regression models, with lower MAE scores meaning a more accurate model and higher *R*^2^ scores meaning a model that can cover a greater variety of data points. CEG was developed to measure the efficacy of BGM systems, and it consists of a grid divided into five zones. Zone A represents values that are clinically accurate and safe, while zones B, C, D, and E represent progressively more significant clinical errors. Typically, only data points within zones A and B are accepted by clinicians. A total of 8 (55%) devices achieved a CEG (A+B) of 100%, all of which included supervised learning models.

Various ML and deep learning (DL) algorithms were used. Nine [25,28,31,36,37,40,42,46,51] devices used only DL models (often some kind of neural network [NN]), while 8 [15,23,24,27,38,39,50,58] devices included only ML models. The rest used a mix of models. Among the DL models or NNs, 6 devices [2,31,40,42,49,56] used artificial neural networks (ANN), and three devices [12,25,54] used deep NNs. Among the ML models, 10 devices [12,14,22,27,34,38,41,44,53,54] used random forest (RF), 8 devices [12,34,41,43,44,49,54,56] used linear regression, 5 devices [14,26,29,34,49] used support vector machines (SVM) and 5 devices [22,41,44,53,54] used support vector regression. Datasets were split according to ratios ranging from 70:30 to the traditional 80:20 for training and testing according to different studies.

The most popular ML algorithm used was RF. Incidentally, RF is widely recognized as one of the most effective machine learning algorithms for classification tasks [22]. Increasing the number of trees in the forest improves prediction accuracy, allowing for tailored models based on specific characteristics. One study which used the use of RF had an accuracy of 94.2% [45], while another study that examined the use of RF to predict HbA_1c_ achieved a low mean average percent error of 4.87% [49].

Another popular algorithm used for data classification is SVM. SVM uses nonlinear mapping to transform DM training data into a higher dimension and seeks the optimal linear separating hyperplane [22]. It aims to create distinct margins between different classes, improving the training and testing speed. In a study based on salivary electrochemical signals, SVM outperformed other models in estimating blood glucose levels with 85% accuracy, 84% precision, and 85% sensitivity [34]. SVM had the best performance in another study which used PPG signals with an accuracy of 81.7% [44].

NNs are a popular DL model extensively used for the detection and diagnosis of DM. This was evident in a study that used CNN to estimate blood glucose levels using breath signals [32]. Performance was promising with a low mean square error of 0.14 and area under the curves as 0.97, 0.96, and 0.96 for T1DM, T2DM, and healthy, respectively [32]. ANN performed best when using input from the Pima Indian diabetes dataset, achieving an overall accuracy of 88.6% [54].

### Study Quality

**Figure 3:**
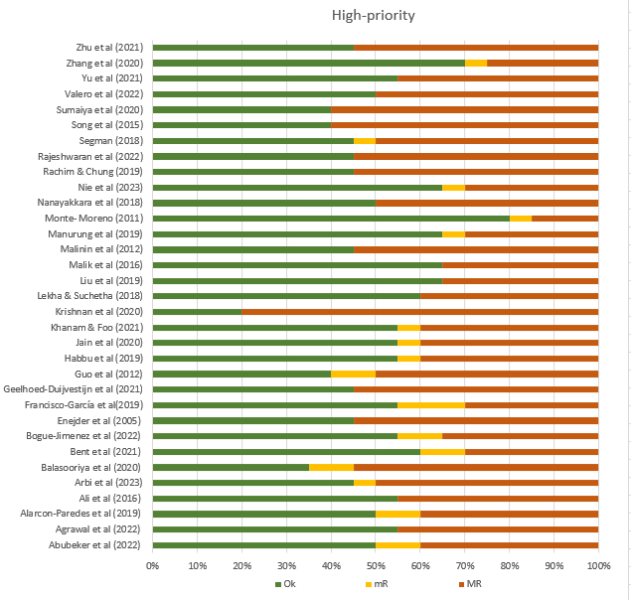
Proportion of OK=adequately addressed, mR=sufficient but improvable, MR= inadequately addressed ratings on each high priority items.

**Figure 4:**
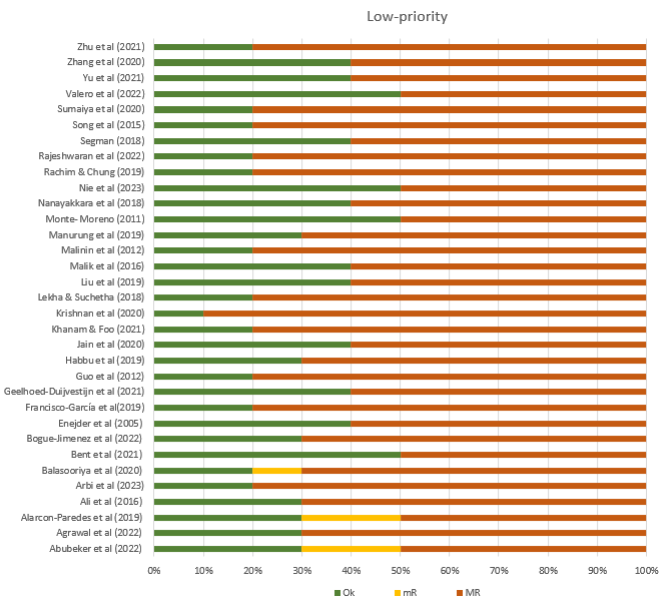
Proportion of OK=adequately addressed, mR=sufficient but improvable MR= inadequately addressed ratings on each low priority items.

The mean overall ChAMAI score on the included papers was 33.5 (SD 3.09), suggesting an average of medium quality (Table 2). Most of the studies were of medium quality ranging between 30 and 41, while 10 studies were of high quality with a score equal to or more than 35. The proportion of “OK,” “mR,” and “MR” in high-priority items range from 20% to 80%, 0% to 6.7%, and 6.7% to 80%, respectively (Figure 3). The proportion of “OK,” “mR,” and “MR” in low-priority items range from 10% to 50%, 0% to 20%, and 50% to 90%, respectively (Figure 4). The interrater agreement in using ChAMAI indicated moderate agreement (Cohen κ=0.49).

**Table 2:** Study quality rated based on ChAMAI^a^.

Author (Year)	Problem understanding (10)	Data understanding (6)	Data preparation (8)	Modeling (6)	Validation (12)	Deployment (8)	Overall (50)
Abubeker and Baskar (2022) [44]	7	4	5	6	7	4	33
Agrawal et al (2022) [10]	8	4	4	6	8	4	34
Alarcón-Paredes et al (2019) [47]	7	3	4	6	10	3	33
Ali et al (2016) [46]	7	4	5	6	9	3	34
Arbi et al (2023) [48]	7	3	5	6	7	4	32
Balasooriya and Nanayakkara (2020) [28]	7	3	4	5	7	3	29
Bent et al (2021) [49]	10	4	4	5	10	4	37
Bogue-Jimenez et al (2022) [50]	7	3	5	6	10	3	34
Enejder et al (2005) [51]	9	4	5	5	8	2	33
Francisco-García et al (2019) [58]	7	4	5	6	8	3	33
Geelhoed-Duijvestijn et al (2021) [52]	10	5	4	5	6	3	33
Guo et al (2012) [30]	7	3	5	6	6	3	30
Habbu et al (2019) [31]	8	4	5	5	9	3	34
Jain et al (2020) [53]	8	4	5	5	10	3	35
Khanam and Foo (2021) [54]	7	3	7	5	8	3	33
Krishnan et al (2020) [45]	5	3	4	5	6	2	25
Lekha and Suchetha (2018) [32]	7	4	5	6	9	3	34
Liu et al (2019) [33]	9	5	4	6	9	4	37
Malik et al (2016) [34]	10	4	5	6	9	3	37
Malinin (2012) [55]	6	3	5	6	9	2	31
Manurung et al (2019) [42]	7	5	5	6	10	3	36
Monte-Moreno (2011) [56]	9	6	7	6	10	3	41
Nanayakkara et al (2018) [29]	9	4	4	5	9	3	34
Nie et al (2023) [35]	9	5	5	6	10	3	38
Rachim and Chung (2019) [36]	6	3	4	6	9	3	31
Rajeshwaran et al (2022) [43]	6	3	5	6	8	3	31
Segman (2018) [37]	9	5	4	6	6	3	33
Song et al (2015) [38]	7	3	4	5	8	3	30
Sumaiya et al (2020) [27]	5	3	5	6	8	3	30
Valero et al (2022) [57]	9	4	4	6	8	4	35
Yu et al (2021) [39]	9	4	5	6	8	3	35
Zhang et al (2020) [40]	9	6	5	6	9	3	38
Zhu et al (2021) [41]	6	4	4	6	8	3	31

^a^ChAMAI: Checklist for assessment of medical artificial intelligence.

## Discussion

### Principal Findings

Findings from this scoping review revealed the applications of AI-assisted NIBGM systems, available technology developed, and types of AI algorithms from the 33 included studies published between 2005 and 2023. Most studies (n=20, 60%) originated from just 3 countries mainly China, India, and the United States.

The bulk of the evidence comes from Asian studies, potentially due to the alarming increase in the prevalence of DM in Asia compared to their European counterparts [60]. There was an even mix of studies from low-, middle- and high-income countries but it is unclear whether AI technologies can be made affordable and accessible to individuals in low- and middle-income countries.

More research can be done to determine the cost and accessibility of AI-assisted glucose monitoring systems and their barriers to widespread adoption. A significant number of studies were reported in conference proceedings, which reflect the emerging evidence regarding AI in NIBGM. Perhaps more research relating to diagnostic accuracy can be conducted to increase the strength of evidence for the adoption of such technology over current traditional glucose monitoring systems.

The majority of studies that develop ML algorithms to predict DM used the Pima Indian diabetes dataset which comprises 8 parameters. These criteria include the number of pregnancies, BMI, plasma glucose concentration, blood pressure, skinfold thickness, diabetes pedigree function, and an outcome variable of class 0 or 1 (where 0 denotes patient without diabetes and 1 denotes patient with diabetes) [61,62]. Other features include waveform characteristics from optical signals, such as shape and amplitude, to estimate blood glucose levels [33,36,40]. AI advances in the field of blood glucose estimation research in the context of NIBGM have the potential to improve the quality of life for patients with DM and minimize invasiveness.

### Application

AI was mainly used in NIBGM to estimate real-time blood glucose levels using optical, biosensing, imaging, and tissue impedance measurement technology instead of current widely used methods such as blood tests or finger pricks [63]. AI was also used to predict future blood glucose levels (up to 30 minutes later) [22] and detect DM [10,44], suggesting the potential of AI-assisted NIBGM for continuous BGM and diagnostic purposes.

### Technology Used

Two broad classifications for NIBGM emerged namely sample- and non–sample-based methods of detection. Sample-based include studies like Malik et al [34] which use salivary electrochemical signals to train ML models. Concentrations of sodium, potassium, and calcium ions were measured and correlated with blood glucose levels [34]. Other sample-based techniques include the use of breath signals to detect acetone to estimate blood glucose levels [30,32]. A major challenge for the development of NIBGM systems which rely on bodily fluid is that the concentration glucose level is miniscule [64]. Hence, there is a need to enhance sensitivity and remove other interference in such sensors [65].

Out of the non–sample-based noninvasive techniques developed to predict blood glucose levels, PPG, akin to the technology of pulse oximetry, appears the most among the studies followed by other optical techniques such as NIRS and RS. The results were not surprising as the use of optical methods for measuring glucose levels is presently one of the best approaches in noninvasive glucose estimation research [47]. For example, Monte-Moreno [56] used a PPG-based sensor to measure changes in blood volume changes and developed an ML algorithm to estimate blood glucose levels [14]. While traditional PPG requires skin contact, typically using a finger over a smartphone camera, to detect blood volume changes, advanced remote PPG allows the detection of subtle skin color changes to estimate blood volume changes [27,35,40]. These technologies have to be validated against the conventional BGM methods in a larger clinical population to establish their usefulness and efficiency [66].

Such technology may be useful for self-monitoring since it has a low barrier of entry and only requires a smartphone. Such setup may also be useful in clinical settings for monitoring or diagnostic purposes and reduces the need for retraining since staff are familiar with similar setups in the hospitals. On the other hand, others have commented that the use of PPG is often corrupted by measurement artifacts from movements, restricting one’s movement during continuous glucose monitoring [36,67].

### ML Models

This review maps out the various DL and traditional ML algorithms used by the studies. Previous studies have adopted ML for risk stratification and identification of patients with DM [68]. Several ML processes, such as SVM, regression trees, k-nearest neighbor, ANN, naïve Bayes, and RF, have been used in transforming diabetes care [69].

The main uses of ML processes include feature selection and classification. ML methods require the extraction of features from signals. However, extracting fiducial points from real-life signals can be highly challenging [21]. Not only is it difficult to develop a feature extraction algorithm that can handle diverse waveform types but there is also a need to assess the quality of the computed features as the feature extraction algorithm is unable to effectively operate if the input signal is corrupted [21].

The emergence of DL has facilitated the analysis of large volumes of data without the need for explicit feature extraction. However, DL approaches experience limited interpretability, which can be problematic in a clinical setting where understanding why and how a pathology was detected is crucial for validating the diagnosis [21].

### Future Research

In different studies, researchers have used various ML algorithms to construct classification models using derived feature vectors to evaluate the performance of different algorithms on the datasets used. Conversely, some researchers have opted to use a single ML method for their classification model. However, it is important to note that no single ML algorithm is universally optimal for all types of input data [70]. Therefore, it is beneficial to test multiple ML algorithms and determine which one produces the best outcomes for a given task. Comparisons among different AI models can help identify the strengths and limitations of each approach, guiding further improvements in accuracy and performance.

Given the heterogeneity of AI models and input data applied in each study, it is beyond the scope of this review to ascertain the best NIBGM system based on performance metrics alone. Furthermore, the lack of standardized reporting and analysis of results, leads to heterogeneity that hampers the comparison of findings across studies. Perhaps a diagnostic accuracy review may be more suited to address the question of which system is best suited to be adopted in various settings. As with all AI studies, efforts should be made to standardize and regulate the use of AI technologies in diabetes care. Consensus guidelines and protocols should be developed to ensure the quality and safety of AI-assisted monitoring systems [71].

Another potential area of research in the field of NIBGM is the use of digital twin (DT) techniques. DT serves as a digital representation that mirrors the state of a physical entity or system by capturing real-time data through sensors and reflecting it in digital devices [72]. DT offers a powerful solution for real-time monitoring, accurate diagnosis, and effective treatment [72]. However, the main challenges include data acquisition, data privacy, and security concerns [73]. Further advancement in Big Data is required to develop holistic and accurate DTs.

### Strengths and Limitations

To the best of our knowledge, this is the first study to report the current state-of-the-art in AI-assisted NIBGM, which informs the direction of future systematic reviews and interventional research. To enhance the rigor of the study, we adhered to the PRISMA-ScR guidelines and had 2 independent reviewers in the paper selection process.

This study had several limitations. First, as this review limits in vivo methods of verification (human participants), certain relevant evidence could have been precluded, such as studies that use in vitro methods for verification of their AI models such as skin models or varied concentrations of glucose solutions. Second, a simple keyword search strategy and only papers written in English were retrieved, possibly limiting the scope of our findings. However, we conducted a hand search of previous systematic reviews to identify relevant papers.

### Conclusions

The use of AI for NIBGM is a promising area of research that has the potential to revolutionize diabetes management. The studies reviewed demonstrate that some AI techniques can accurately predict glucose levels from noninvasive sources while enhancing comfort and ease of use for patients. However, the overall range of accuracy is wide due to the heterogeneity of models and input data. As such, we propose that there is a need for further efforts to standardize and regulate the use of AI technologies in diabetes care, as well as develop consensus guidelines and protocols to ensure the quality and safety of AI-assisted monitoring systems.

## Appendix

Multimedia Appendix 1 PRISMA-ScR (Preferred Reporting Items for Systematic Reviews and Meta‐Analyses extension for Scoping Reviews) checklist.

Multimedia Appendix 2 Specific search strategy for each database.

Multimedia Appendix 3 Characteristics of blood glucose monitoring systems.

Multimedia Appendix 4 Characteristics of noninvasive blood glucose monitoring systems.

Multimedia Appendix 5 Characteristics of Machine Learning.

## Abbreviations

AI: artificial intelligence
ANN: artificial neural network
BGM: blood glucose monitoring
CEG: Clarke error grid
ChAMAI: checklist for assessment of medical AI
DL: deep learning
DM: diabetes mellitus
DT: digital twin
ECG: electrocardiography
HbA1c: hemoglobin A1c
MAE: mean absolute error
ML: machine learning
NIBGM: noninvasive blood glucose monitoring
NIRS: near-infrared spectroscopy
NN: neural network
PPG: photoplethysmography
PRISMA-ScR: Preferred Reporting Items for Systematic Reviews and Meta‐Analyses extension for Scoping Reviews
RF: random forest
RS: Raman spectroscopy
SVM: support vector machines
UWB: ultrawideband

## Data Availability

All data generated or analyzed during this study are included in this published article.
