# Supplementary material for: AI-Based Noninvasive Blood Glucose Monitoring: Scoping Review
Source: J Med Internet Res. 2024 Nov 19;26:e58892. doi: 10.2196/58892 (PMC11615544; doi:10.2196/58892)
Supplement: Multimedia Appendix 2 [file jmir_v26i1e58892_app2.docx]

| **Database** | **Index and keyword terms** | **Results** |
| --- | --- | --- |
| *PubMed* | ("glucose monitoring"[Title/Abstract] OR "monitoring glucose"[Title/Abstract]) AND (artificial intelligence[Title/Abstract] OR computer heuristics[Title/Abstract] OR fuzzy logic[Title/Abstract] OR knowledge bases[Title/Abstract] OR machine learning[Title/Abstract] OR natural language processing[Title/Abstract] OR neural networks[Title/Abstract] OR sentiment analysis[Title/Abstract]) | 135 |
| *EMBASE* | ('glucose monitoring' OR 'monitoring glucose') AND ('artificial intelligence':ti,ab,kw OR 'computer heuristics':ti,ab,kw OR 'fuzzy logic':ti,ab,kw OR 'knowledge bases':ti,ab,kw OR 'machine learning':ti,ab,kw OR 'natural language processing':ti,ab,kw OR 'neural networks':ti,ab,kw OR 'sentiment analysis':ti,ab,kw) | 375 |
| *The Cochrane Library (CENTRAL-trials only)* | “glucose monitoring” OR “monitoring glucose” in Title Abstract Keyword AND artificial intelligence OR computer heuristics OR fuzzy logic OR knowledge bases OR machine learning OR natural language processing OR neural networks OR sentiment analysis in Title Abstract Keyword - (Word variations have been searched) | 128 |
| *CINAHL* | AB ( “glucose monitoring” OR “monitoring glucose” ) AND AB ( artificial intelligence OR computer heuristics OR fuzzy logic OR knowledge bases OR machine learning OR natural language processing OR neural networks OR sentiment analysis ) | 22 |
| *Scopus* | ( TITLE-ABS-KEY ( "glucose monitoring" OR "monitoring glucose" ) AND ALL ( artificial AND intelligence OR computer AND heuristics OR fuzzy AND logic OR knowledge AND bases OR machine AND learning OR natural AND language AND processing OR neural AND networks OR sentiment AND analysis ) ) | 2 |
| *Web of Science* | “glucose monitoring” OR “monitoring glucose” (Topic) and artificial intelligence OR computer heuristics OR fuzzy logic OR knowledge bases OR machine learning OR natural language processing OR neural networks OR sentiment analysis (Topic) | 389 |
| *IEEE Xplore* | ("Abstract":“glucose monitoring” OR "Abstract":“monitoring glucose”) AND ("Abstract":artificial intelligence OR "Abstract":computer heuristics OR "Abstract":fuzzy logic OR "Abstract":knowledge bases OR "Abstract":machine learning OR "Abstract":natural language processing OR "Abstract":neural networks OR "Abstract":sentiment analysis) | 202 |
| *ACM digital library* | [[Abstract: artificial intelligence] OR [Abstract: computer heuristics] OR [Abstract: fuzzy logic] OR [Abstract: knowledge bases] OR [Abstract: machine learning] OR [Abstract: natural language processing] OR [Abstract: neural networks] OR [Abstract: sentiment analysis]] AND [[Abstract: "glucose monitoring"] OR [Abstract: "monitoring glucose"]] | 17 |
