# Supplementary material for: AI-Based Noninvasive Blood Glucose Monitoring: Scoping Review
Source: J Med Internet Res. 2024 Nov 19;26:e58892. doi: 10.2196/58892 (PMC11615544; doi:10.2196/58892)
Supplement: Multimedia Appendix 3 [file jmir_v26i1e58892_app3.docx]

| Author (Year) | Usage | AI type | AI features | Input data | Technology | Dataset | Validation | Training/Testing | Metrics used |
| --- | --- | --- | --- | --- | --- | --- | --- | --- | --- |
| Abubeker and Baskar (2022) [56] | Estimate BG levels | SVM, KNN | Body mass index, blood pressure, BG history, age, skin thickness, and clinical outcome | SpO2, HR, ambient temperature | PPG | PIMA Indian diabetes datasets + Kaggle | external | 973 training, 15 validation | Accuracy, Precision, Recall , F1, MSE |
| Agrawal et al. (2022) [10] | Estimate BG levels | LR, KNN, DT, RF, GB, GNB, DNN | Number of Times Pregnant, Plasma glucose concentration, Diastolic blood Pressure (mm Hg), Skin Fold Thickness(mm) , 2-Hour serum insulin(U/ml), BMI (weighted gl(height∕mm), Diabetes pedigree function, Age in Years, Class ’0’ or ’1 | NIR signals | NIR spectroscopy | PIMA Indian diabetes datasets/ iGLU dataset | NR | 80% training, 20% testing | MAD, MARD, RMSE, CEG |
| Alarcon-Paredes et al. (2019) [30] | Estimate BG levels | ANN | NR | Visible light signals | Non-invasive optical analysis | NR | 10-fold cross-validation | 70% test/train, 30% validation | CEG, MAE |
| Ali et al. (2016) [58] | Estimate BG levels | ANN | Characteristic features where only four feature values like standard deviation, mean, maxima and minima were considered | UWB imaging | UWB imaging | UniMAP students and local volunteers | external | 70% training, 15% testing, 15% validation | Accuracy |
| Arbi et al., 2023 [31] | Estimate BG levels | LR, NLR, EGPR | T-wave, QRS, P-wave intervals, T-wave amplitude, QTc, and HR | ECG signal | NR | NR | NR | NR | RMSE, R^2^, MAE |
| Balasooriya and Nanayakkara (2020) [28] | Predict the BG level 30 minutes in the future | LSTM | body, weight, height, age, gender | Medication intake, food intake, daily activities and measured blood glucose levels | NR | NR | NR | NR | Accuracy, RMSE |
| Bent et al. (2021) [32] | Predict HbA1c | RF | GMI, Interday Mean Glucose, Interday Median Glucose, Interday Quartile 1 Glucose, Interday Quartile 3 Glucose, Mean of Glucose Excursions (MGE), Mean of Intraday SD, SD of Intraday SD, Time Inside Range (TIR), Per cent Time Inside Range, and Mean of Normal Glucose | skin temperature, electrodermal activity, accelerometry, HR | NR | NR | External | Separate validation cohort | RMSE, MAPE |
| Bogue-Jimenez et al. (2022) [33] | Estimate BG levels | LR, SVR, KNN, DTR, BTR, RFR, GPR, MLP | Ambient temp, HR, Skin temp, Glavanic skin response | Ambient temperature, HR, Skin temperature, Glavanic skin response | Optical, electromagnetic, and thermal techniques | Ohio dataset | 10-fold cross-validation | 75% training, 25% testing | RMSE, CEG |
| Enejder et al. (2005) [34] | Estimate BG levels | PLS | NR | Raman spectra | Raman spectroscopy | NR | leave-one-out cross validation | NR | MAE, R^2^ |
| Francisco-García et al. (2019) [53] | Estimate BG levels | LR, KNN, RF. SVR, RT | Mel frequency cepstral coefficients (MFCC) | Light signal | laser beam & LDR | NR | 5-fold cross-validation | NR | MAE, CEG |
| Geelhoed-Duijvestijn et al. (2021) [35] | Estimate BG levels | NNR | NR | Tears | Biosensor for tear glucose | Haaglanden Diabetes Centre | NR | NR | MARD, MedARD, CEG |
| Guo et al. (2012) [36] | Estimate BG levels | SVOR | NR | Breath signal | Breath signal analysis | 110 outpatient, 82 inpatient | NR | NR | Accuracy |
| Habbu et al. (2019) [37] | Estimate BG levels | Neural network | PPG waveform, Kaiser Teager Energy, HR, Spectral entropy, energy profile, peak to peak interval | PPG signal | PPG | Jahangir Medical and Research Centre, Freedom from Diabetes Organization India, Vishwakarma Institute of Information Technology | cross validation | NR | R^2^, CEG |
| Jain et al. (2020) [38] | Estimate BG levels | DNN | NR | Light signals | NIR spectroscopy | NR | external | 97 calibration, 93 validation and testing | MARD, AvgE, MAD, RMSE CEG |
| Khanam & Foo (2021) [39] | Estimate BG levels | DT, KNN, RF, NB, AB, LR, SVM, NN | Pregnancy, BMI, insulin level, age, BP, skin thickness, glucose, diabetes pedigree function, outcome | Attributes from dataset | NR | Pima Indian dataset | k cross validation method | 85% training, 15% testing | Accuracy |
| Krishnan et al. (2020) [57] | Estimate BG levels | RF | NR | PPG signals | PPG | NR | NR | NR | Accuracy |
| Lekha & Suchetha (2018) [40] | Estimate BG levels | CNN | NR | Signals from gas sensors |  | NR | leave-one-out cross validation | 15 training, 10 testing | AUC, MSE |
| Liu et al. (2019) [41] | Estimate BG levels | PLS, SVR, ANN, RF, Ada | Temperature, pressure diff signal, pulse wave signal, shape | Light signals | Absorption spectroscopy | PLA Navy General Hospital | 10-fold cross-validation | 50 training, 39 testing | R^2^, MAE, MSE, MRAE, precision, recall, F1, AUC, CEG |
| Malik et al. (2016) [42] | Estimate BG levels | LR, SVM, ANN | pH, redox potential, conductivity and concentration of sodium, potassium and calcium ions | Salivary electrochemical signals |  | recruited volunteers | cross validation | 70% training, 30 % testing | Accuracy, precision, sensitivity, F1 score |
| Malinin et al. (2012) [43] | Estimate BG levels | Neural network | High frequency impedance, low frequency impedance, skin temperature, time, food intake, beverage intake, insulin, physical activity | Impedance data | Impedance | NR | NR | NR | CEG |
| Manurung et al. (2019) [54] | Estimate BG levels |  | NR | LED signals | NIR spectroscopy | NR | NR | 40 training, 11 testing | MAE |
| Monte-Moreno (2011) [44] | Estimate BG levels | RLR, MPNN, SVM, RF | KTE, HR, O2 saturation range, Spectral entropy, | PPG signals | PPG | Universitat Politecnica de Catalunya & ambulatory primary care centre | 10-fold cross validation | NR | R^2^ |
| Nanayakkara et al. (2018) [29] | Estimate BG levels | ANN, LR | Body temperature, weight | NIR signals | NIR spectroscopy, bio-impedance | NR | internal | 40 training, 30 testing | CEG |
| Nie et al. (2023) [45] | Estimate BG levels | PCR, PLS, SVR, RFR | 6 from the time-domain (e.g., height, time, area, etc.), energy-domain and human physiological parameters | Facial video | PPG | School of Physics and Technology, Wuhan University | 10-fold cross-validation | 80% training, 20% testing |  |
| Rachim & Chung (2019) [46] | Estimate BG levels | PLS | Amplitude of PPGAC component in 950, 850, 660, 530 nm, Amplitude of component in 950, 850, 660, 530 nm, Difference of Optical Density in 950, 850, 660, 530 nm, Variance, skewness, SD of TKEO | PPG signals | PPG | NR | 10-fold cross validation | NR | R_p_, SEP, CEG |
| Rajeshwaran et al. (2022) [55] | Estimate BG levels | DT, XG boost, SVM, NB | Glucose, HR, temp | Sensor values | NR | NR | NR | 80% training, 20% testing | Accuracy |
| Segman (2018) [47] | Estimate BG levels | NBN | NR | Optical signals | Colour image sensor | NR | NR | NR | CEG |
| Song et al. (2015) [48] | Estimate BG levels | ANN | NR | C_p_ from IMPS and three I_pds_ from mNIRS | Impedance and Multi-Wavelength NIR Spectroscopy | NR | NR | 15 samples training, 17 verification, 17 testing | CEG |
| Sumaiya et al. (2020) [27] | Estimate BG levels | LR, SVR, DNN, RFR | PPG waves | Video data of finger over smartphone camera | PPG | NR | 10-fold cross-validation | NR | R, R^2^, MAE, MSE, RMSE, MSLE, EVS |
| Valero et al. (2022) [49] | Estimate BG levels | CNN | NR | Finger/ear images |  | NR | NR | 80% training, 20% testing | Accuracy |
| Yu et al. (2021) [50] | Estimate BG levels | PLS ElM | NR | Optical signals | NIR | volunteers | cross validation | NR | RMSE, CEG |
| Zhang et al. (2020) [51] | Estimate BG levels | GSVM | 28 features (H1, H2, n1, n2, W1, W2, highest_peak, dis_peak, notchtime_notch, timediff_peak_notch, timediff_notch_diastolicpeak, timediff_diastolicpeak_end, area_single, area_start_max, area_max_notch, area_notch_diastolicpeak, area_diastolicpeak_end | 60-s video of the left index finger | PPG | Qilu Hospital of Shandong University | NR | 40 training, 40 testing | Accuracy |
| Zhu et al. (2021) [52] | Estimate BG levels | BPNN | NR | Metabolic heat production (H), HR, SpO2, and blood flow rate (BF) | NR | NR | NR | 118 training, 93 testing | MARD, MAD, RMSE, SEP, CEG |

Abbreviation: ANN=Artificial Neural Network; AUC=Area Under Curve; CEG=Clarke Error Grid; DNN=Deep Neural Network; DT=Decision Tree; EGPR=Exponential Gaussian Process Regression; EVS=Enumerator variances; GB=Gradient Boost; GNB=Gaussian Naïve Bayes; HR=Heart Rate; MAD= Mean Absolute Deviation; MAE=Mean Absolute Error; MAPE=Mean Average Percent Error; MARD=Mean Absolute Relative Difference; MSE=Mean Square Error; MSLE=Mean Square Logarithmic Error; NB=Naïve Bayes; NIR=Near Infra-red; NR=Not Reported KNN=K Nearest Neighbour; LR=Linear Regression; NLR=Non Linear Regression; PPG= Photoplethysmography; R=Correlation Coefficient; R^2^=Correlation of Determination; RF=Random Forest; RMSE=Root Mean Square error; SEP=;SVM=Support Vector Machine
