# Supplementary material for: AI-Based Noninvasive Blood Glucose Monitoring: Scoping Review
Source: J Med Internet Res. 2024 Nov 19;26:e58892. doi: 10.2196/58892 (PMC11615544; doi:10.2196/58892)
Supplement: Multimedia Appendix 4 [file jmir_v26i1e58892_app4.docx]

| Author (Year) | Population Characteristics | Name of device | Use case | Input data | Technology | Mechanism |  |
| --- | --- | --- | --- | --- | --- | --- | --- |
| Abubeker and Baskar (2022) [56] | NR | NR | Estimate BG levels and predict suitable insulin levels | SpO^2^, HR, ambient temperature | PPG | PPG signal using MAX30102 sensor and calibration of the blood flow fluctuation with reference to temperature using MLX90614. ML techniques are used in the second half of the system to anticipate blood glucose levels and recommend insulin dosages based on a patient's history from their local database. |  |
| Agrawal et al. (2022) [10] | NR | Intelligent glucometer device iGLU | Detect DM and estimate BG levels | NIR signals | NIR spectroscopy | Data is accumulated by placing three fingers between the emitter and detector lined with the pads. |  |
| Alarcon-Paredes et al. (2019) [30] | Healthy | Raspberry Pi Zero | Estimate BG levels | Visible light signals | Non-invasive optical analysis | Laser-beam is passed through the finger as a medium while a camera acts as the sensor to capture the transmitted light and how it is scattered along the finger. |  |
| Ali et al. (2016) [58] | NR | UWB Micro strip patch bio-antenna | Estimate BG levels | UWB imaging | UWB imaging | Antenna transmits UWB pulse train through left hand mussel, forward scattered signals were received by Rx antenna from the other side. |  |
| Arbi et al., (2023) [31] | QT Dataset: NR  D1NAMO database: NR; 9 Type 1 DM | NR | Estimate BG levels | ECG signal | NR | ECG signal was transformed using Fast Fourier Transform, and a band-pass filter to remove artefacts. ECG was segmented using convolutional neural network. |  |
| Balasooriya and Nanayakkara (2020) [28] | 3 DM | NR | Predict the BG level 30 minutes in the future | Medication intake, food intake, daily activities and measured blood glucose levels | NR | Features including subject height, weight, age, gender were used to estimate blood glucose level. |  |
| Bent et al. (2021) [32] | Dataset 1: 8 pre-DM, 8 high-normal glucose  Dataset 2:  5:5; 5 pre-DM, 5 high-normal glucose | \| Wrist worn wearable Empatica E4 \| \| --- \| \|  \| | Predict HbA1c and glucose variability | Skin temperature, electrodermal activity, accelerometery, HR | NR | Used data from the wearable to develop ML models to predict HbA1c recorded on day 0 and glucose variability calculated from the CGM. |  |
| Bogue-Jimenez et al. (2022) [33] | 12 Type 1 DM | Basis Peak, Empatica E4 | Estimate BG levels | Ambient temperature, HR, Skin temperature, galvanic skin response | Optical, electromagnetic, and thermal techniques | Multiple off-the-shelf wearable sensors and learning-based models to predict blood glucose. |  |
| Enejder et al. (2005) [34] | All Healthy | NR | Estimate BG levels | Raman spectra | Raman spectroscopy | Diode lasers emit beam which pass through a band pass filter towards a paraboloidal mirror by a small prism and focused onto human forearm, backscattered Raman light was collected by the mirror and passed through a notch filter. |  |
| Francisco-García et al. (2019) [53] | NR | NR | Estimate BG levels | Light signal | Laser beam & light diode resistor (LDR) | LDR was used as a photodiode to measure the light transmitted by a 650 nm wavelength laser-beam across the finger. |  |
| Geelhoed-Duijvestijn et al. (2021) [35] | 24 Type 1 DM | NovioSense | Estimate BG levels | Tears | Biosensor for tear glucose | Sensors were placed into the fornix of the lower eyelid. |  |
| Guo et al. (2012) [36] | 123 Type 2 DM | NR | Estimate BG levels | Breath signal | Breath signal analysis | Sensors were used to detect acetone. |  |
| Habbu et al. (2019) [37] | 233 DM | NR | Estimate BG levels | PPG signal | PPG | PPG signals were recorded by a Pulsed Data Acquisition module. |  |
| Jain et al. (2020) [38] | 52 Pre-DM, 58 DM | Intelligent Glucose Meter | Estimate BG levels | NIR signals | NIR spectroscopy | Short NIR waves with absorption and reflectance of light using specific wavelengths (940 and 1,300 nm) were used. |  |
| Khanam & Foo (2021) [39] | NR | NR | Detect diabetes mellitus | Pregnancy, BMI, Insulin level, Age, Blood pressure, Skin thickness, Glucose, Diabetes pedigree function | NR | NR |  |
| Krishnan et al. (2020) [57] | NR | NR | Estimate BG levels | PPG signals | PPG | Detect blood volumetric changes by pressure pulse detected by transmitting NIR light into the skin and measuring the amount of transmitted or reflected light by the photodiode. |  |
| Lekha & Suchetha (2018) [40] | 9 Type 2 DM, 5 Type 1 DM | NR | Detect diabetes mellitus | Signals from gas sensors | NR | Electro-chemical sensors with good affinity to volatile organic compounds in breath. |  |
| Liu et al. (2019) [41] | NR | Earlight | Estimate BG levels | Light signal | Absorption spectroscopy | Four narrow bandwidth Light-emitting Diodes of different wavelengths transmits light through the earlobe and a light intensity sensor that measures the intensity of the light transmitted. |  |
| Malik et al. (2016) [42] | 88 T1 DM | NR | Estimate BG levels | Salivary electrochemical signals | NR | Saliva samples were obtained and used to train ML |  |
| Malinin et al. (2012) [43] | NR | NR | Estimate BG levels | Impedance data | Impedance | Glucose estimates, based on high frequency skin impedance, measured by tetrapolar electrodes. |  |
| Manurung et al. (2019) [54] | NR | NR | Estimate BG levels | LED signals | NIR spectroscopy | NIR gets absorbed by glucose molecules in the dermis layer, reflected intensity will get increased in diabetic pts |  |
| Monte- Moreno (2011) [44] | NR | iPod Digital Oximeter | Estimate BG levels | PPG signals | PPG | Using ML to predict glucose levels through shape of PPG waveform |  |
| Nanayakkara et al. (2018) [29] | NR | NR | Estimate BG levels | NIR signals | NIR spectroscopy, bio-impedance | 940nm peak intensity wavelength IR transmitter and 940nm peak sensitivity photo diode for the transducer to estimate the absorption due to glucose |  |
| Nie et al. (2023) [45] | NR | NR | Estimate BG levels | Facial video | PPG | A near-infrared camera captures a video of the subject’s face and transmits the video to the computer for subsequent analysis. |  |
| Rachim & Chung (2019) [46] | NR | NR | Estimate BG levels | NIR signals | NIR spectroscopy | A multi-chip sensor package was used to transmit infrared LED light of 950 nm, Red LED light of 660 nm, and Green LED light of 530 nm, and to detect the reflected light by using an integrated photodiode with a spectral working range of 400–1100 nm. |  |
| Rajeshwaran et al. (2022) [55] | Unclear | ESP8266-12E wi-fi module controller | Estimate BG levels | Sensor values | NR | Heart rate and temperature sensor values are collected, sent to the cloud in real-time and trained to estimate BG. |  |
| Segman (2018) [47] | NR | TensorTip Combo Glucometer (CoG) | Estimate BG levels | light signals | Colour image sensor | Real-time colour image sensor analyses capillary blood tint over spatial-temporal-colour domain. |  |
| Song et al. (2015) [48] | NR | NR | Estimate BG levels | C_p_ from IMPS and three I_pds_ from mNIRS | IMPS | IMPS frequency sweep current injector provides sinusoidal current through an off-chip series inductor with bipolar electrode, mNIRS circuit contains three NIR LED drivers to emit three wavelengths (850 nm, 950 nm, and 1,300 nm) NI light and trans-impedance amplifier to detect the intensity of the reflected light from the photo diode. |  |
| Sumaiya et al. (2020) [27] | NR | NR | Estimate BG levels | Video data of finger over smartphone camera | PPG | PPG generated from fingertip video was used. |  |
| Valero et al. (2022) [49] | NR | GlucoCheck | Estimate BG levels | Finger/ear images using visible light | NIR spectroscopy | Raspberry Pi camera captures one image every 8 seconds over 2 minutes, for a total of 15 images. |  |
| Yu et al. (2021) [50] | All no history of hypertension | NR | Estimate BG levels | NIR signals | NIR | Blood glucose levels affect the light intensity reflected from the blood vessel. The higher glucose concentration results in less scattering and hence more absorption, whereas less glucose leads to more scattering and thus less absorption by the tissues |  |
| Zhang et al. (2020) [51] | 50 Hyperglycaemic | NR | Estimate BG levels | 60-s video of the left index finger | PPG | Left-hand index finger video was recorded using a smartphone and PPG signals were extracted from the video |  |
| Zhu et al. (2021) [52] | NR | NR | Estimate BG levels | Metabolic heat production, HR, SpO2, and blood flow rate | NR | NR |  |

Abbreviations: DM=diabetes mellitus; IMPS=Impedance and Multi-Wavelength NIR Spectroscopy; NR=not reported; HR=heart rate; PPG=Photoplethysmography; ML=machine learning; NIR=near infrared; LDR=light diode resistor; multi- mNIRS=wavelength near-infrared spectroscopy; ECG=Electrocardiogram; UWB=Ultra-wideband; SpO2=; C_p_
