# Supplementary material for: AI-Based Noninvasive Blood Glucose Monitoring: Scoping Review
Source: J Med Internet Res. 2024 Nov 19;26:e58892. doi: 10.2196/58892 (PMC11615544; doi:10.2196/58892)
Supplement: Multimedia Appendix 5 [file jmir_v26i1e58892_app5.docx]

| Author (Year) | AI Models | AI features | Dataset | Training/Testing | Measure of Accuracy + Value |
| --- | --- | --- | --- | --- | --- |
| Abubeker and Baskar (2022) [56] | SVM, KNN | Body mass index, blood pressure, BG history, age, skin thickness, and clinical outcome | PIMA Indian diabetes datasets + Kaggle | 973 training, 15 validations | SVM: Acc = 0.817, Precision = 0.82, Recall = 0.94, F1 = 0.87, MSE = 4.87 |
| Agrawal et al. (2022) [10] | LR, KNN, DT, RF, GB, GNB, DNN | Number of Times Pregnant, Plasma glucose concentration, Distolic blood Pressure (mm Hg), Skin Fold Thickness(mm), 2-Hour serum insulin(U/ml), BMI (height∕mm), Diabetes pedigree function, Age in Years, Class ’0’ or ’1 | PIMA Indian diabetes datasets/ iGLU dataset | 80% training, 20% testing | MAD = 6.82mg/fdl, mARD = 10.64%, RMSE = 9.14mg/dl, CEG A+B = 100% |
| Alarcon-Paredes et al. (2019) [30] | ANN | NR | NR | 70% test/train, 30% validation | CEG = 90.32% in zone A, MAE = 10.37 |
| Ali et al. (2016) [58] | ANN | Characteristic features where only four feature values likestandard deviation, mean, maxima and minima were considered | UniMAP students and local volunteers | 70% training, 15% testing, 15% validation | Acc = 81% |
| Arbi et al., (2023) [31] | LR, NLR, EGPR | T-wave, QRS, P-wave intervals, T-wave amplitude, QTc, and HR | NR | NR | NR |
| Balasooriya et al. (2020) [28] | LSTM (ANN) | Glucose absorption, energy expenditure during time period, Total drug absorption during time period | NR | NR | Acc = 79.97%, RMSE = 22.2 |
| Bent et al. (2021) [32] | RF | Glucose Management Indicator (GMI), Interday Mean Glucose, Interday Median Glucose, Interday Quartile 1 Glucose, Interday Quartile 3 Glucose, Mean of Glucose Excursions (MGE), Mean of Intraday SD, SD of Intraday SD, Time Inside Range (TIR), Percent Time Inside Range, and Mean of Normal Glucose | NR | Separate validation cohort | MAPE (glucose variability): 11 of the glucose variability metrics was estimated with high performance (MAPE <10%). RMSE ranged from 4.14±2.97% to 162.79±216.70%  MAPE(HbA1c): 4.87%  RMSE(HbA1c): 0.281 |
| Bogue-Jimenez et al. (2022) [33] | LR, SVR, KNN, DTR, BTR, RFR, GPR, MLP | Ambient temp, HR, Skin temp, Galvanic skin response | Ohio dataset | 75% training, 25% testing | BTR: CEG A+B: 86.91%, 93.74% RMSE: 66.32, 46.38, R^2^: 0.11, 0.16 |
| Enejder et al. (2005) [34] | PLS | NR | NR | NR | SVR: MAE=9.45 ± 0.51, CEG(A+B):100%  LR: MAE= 11.4 ± 1.03  RFR: CEG(A+B):100% |
| Francisco-García et al. (2019) [53] | LR, KNN, RF. SVR, RT | Mel frequency cepstral coefficients (MFCC) | NR | NR | SVR: MAE = 9.45 ± 0.51, CEG A + B = 100% |
| Geelhoed-Duijvestijn et al. (2021) [35] | NNR | NR | Haaglanden Diabetes Centre | NR | MARD = 16.7, MedARD = 13.3, CEG A+B = 99.7% |
| Guo et al. (2012) [36] | SVOR | 67 Well-controlled blood glucose levels, 41 Somewhat controlled, 39 Poorly controlled and 45 Not controlled | 110 outpatient, 82 inpatient | NR | Level 1(Well controlled blood glucose levels): SVM: 67.16%, SRC: 65.67%, SVOR: 68.66%  Level 2(Somewhat controlled): SVM: 53.66%, SRC: 56.1%, SVOR: 60.98%  Level 3(Poorly controlled): SVM: 53.85%, SRC: 56.41%, SVOR: 64.10%  Level 4(Not controlled): SVM: 35.56%, SRC: 44.44%, SVOR: 53.33% |
| Habbu et al. (2019) [37] | Neural network | PPG waveform, Kaiser Teager Energy, HR, Spectral entropy, energy profile, peak to peak interval | Jahangir Medical and Research Centre, Freedom from Diabetes Organization India, Vishwakarma Institute of Information Technology | NR | R^2^ = 0.91, CEG A+B= 100% |
| Jain et al. (2020) [38] | DNN | NR | NR | 97 calibration, 93 validation and testing | mARD = 7.32, AvgE = 7.03, MAD = 09.89, RMSE = 11.56, CEG = 100 |
| Khanam & Foo (2021) [39] | DT, KNN, RF, NB, AB, LR, SVM, NN | Pregnancy, BMI, insulin level, age, BP, skin thickness, glucose, diabetes pedigree function, outcome | Pima Indian dataset | 85% training, 15% testing | Acc=88.57% |
| Krishnan et al. (2020) [57] | RF | NR | NR | NR | Acc=94.2% |
| Lekha & Suchetha (2018) [40] | CNN | NR | NR | 15 training, 10 testing | ROC curve with the area under the curves as 0.9659, 0.9625 and 0.9644 for type 1, type 2 and healthy respectively, misclassification rate of 0.0714 and a mean square error of 0.1436 |
| Liu et al. (2019) [41] | PLS, SVR, ANN, RF, Ada | Temperature, pressure diff signal, pulse wave signal, shape | PLA Navy General Hospital | 50 training, 39 testing | ANN: R^2^ = 0.851, MAE = 1.455, MSE = 3.802, RF: MRAE = 0.157, precision = 0,972, recall = 0.937, F1 = 0.955, AUC = 0.980, CEG A+B = 97.9% |
| Malik et al. (2016) [42] | LR, SVM, ANN | pH, redox potential, conductivity and concentration of sodium, potassium and calcium ions | recruited volunteers | 70% training, 30 % testing | SVM: 85 % accuracy, 84 % precision, 85 % sensitivity and 85 % F1 score |
| Malinin et al. (2012) [43] | Neural network | High frequency impedance, low frequency impedance, skin temperature, time, food intake, beverage intake, insulin, physical activity | NR | NR | CEG (A+B) = 92% |
| Manurung et al. (2019) [54] | NR | NR | NR | 40 training, 11 testing | MAE = 5.855 |
| Monte- Moreno (2011) [44] | RLR, MPNN, SVM, RF | Kaiser–Teager energy (KTE), HR, O2 saturation range, Spectral entropy, | Universitat Politecnica de Catalunya & ambulatory primary care centre | NR | RF: R^2^ = 0.90 |
| Nanayakkara et al. (2018) [29] | ANN, LR | Body temperature, weight | NR | 40 training, 30 testing | CEG (A+B) = 100 |
| Nie et al. (2023) [45] | PCR, PLS, SVR, RFR | 6 from the time-domain (e.g., height, time, area, etc.), energy-domain and human physiological parameters | School of Physics and Technology, Wuhan University | 80% training, 20% testing | NR |
| Rachim & Chung (2019) [46] | PLS | Amplitude of  PPGAC component in 950, 850, 660, 530 nm, Amplitude of component in 950, 850, 660, 530 nm, Difference of Optical Density in 950, 850, 660, 530 nm, Variance, skewness, SD of TKEO | NR | NR | R_p_ = 0.86, SEP = 6.16 mg/dL, CEG A = 100% |
| Rajeshwaran et al. (2022) [55] | DT, XG boost, SVM, NB | Glucose, HR, temp | NR | 80% training, 20% testing | XG Boost: Acc = 91.35 |
| Segman (2018) [47] | NBN | NR | NR | NR | \| CEG A+B =100% \| \| --- \| \| CEG A+B =100% \| \| CEG A+B =98.8%, MARD = 17.1% \| |
| Song et al. (2015) [48] | ANN | NR | NR | 15 samples training, 17 verification, 17 testing | CEG A + B = 100% |
| Sumaiya et al. (2020) [27] | LR, SVR, DNN, RFR | PPG waves | NR | NR | DNN: R = 0.927 R^2 = 0.835, MAE = 0.248, MSE = 0.156, RMSE = 0.401, MSLE = 0.002, EVS = 0.835 |
| Valero et al. (2022) [49] | CNN | NR | NR | 80% training, 20% testing | finger acc = 0.79, ear acc = 0.62 |
| Yu et al. (2021) [50] | PLS, ElM | NR | volunteers | NR | CEG (A+B): 100%  PLS:  Rc^2= 0.8963, RMSEC= 0.265, Rp^2= 0.8317, RMSEP= 0.287  EIM:  Rc^2= 0.9157, RMSEC= 0.239, Rp^2= 0.9351, RMSEP= 0.186 |
| Zhang et al. (2020) [51] | GSVM | 28 features (H1, H2, n1, n2, W1, W2, highest_peak, dis_peak, notchtime_notch, timediff_peak_notch, timediff_notch_diastolicpeak, timediff_diastolicpeak_end, area_single, area_start_max, area_max_notch, area_notch_diastolicpeak, area_diastolicpeak_end | Qilu Hospital of Shandong University | 40 training, 40 testing | Acc = 81.49 |
| Zhu et al. (2021) [52] | BPNN | NR | NR | 118 training, 93 testing | mARD = 5.453%, CORR = 0.936, MAD = 1.084mmol/L, RMSE = 0.505mmol/L, SEP = 0.159mmol/L, CEG A = 98.413%, CEG A+B = 100% |

Abbreviations: SVM=Support Vector Machine; KNN=K Nearest Neighbour; BG=blood glucose; Acc=Accuracy, MSE=Mean Square Error; CEG=Clarke Error Grid; LR=Linear Regression; NR=Not Reported;DT=Decision Tree; RF=Random Forest; GB=Gradient Boost; GNB=Gaussian Naïve Bayes; DNN=Deep Neural Network; ANN=Artificial Neural Network; NLR=Non Linear Regression; EGPR=Exponential Gaussian Process Regression; LSTM=Long short-term memory; RMSE=Root Mean Square error; MAPE=Mean Average Percent Error; SVR=support vector regression; DTR=dynamic treatment regime; DTR=decision tree regression; R=Correlation Coefficient; R^2^=Correlation of Determination; SEP= standard error of prediction BTR=bagging trees regression; RFR=Random Forest Regression; GRP=Gaussian process regression; MLP=Multi-layer Perceptron Regression; PLS= Partial least squares; NNR= Neural Network Regression; MARD=Mean Absolute Relative Difference; SVOR=Support vector ordinal regression; AUC=Area Under Curve; CNN=convolutional neural network; EVS=Enumerator variances; HR=Heart Rate; MAD= Mean Absolute Deviation; MAE=Mean Absolute Error; MSLE=Mean Square Logarithmic Error; NB=Naïve Bayes; NIR=Near Infra-red; PPG= Photoplethysmography; QT Database=Database of QT wave
